# Dataset on assessment of pollution level of selected trace metals in farming area within the proximity of a gold mine dump, Ekuhurleni, South Africa

**DOI:** 10.1016/j.dib.2019.104473

**Published:** 2019-09-03

**Authors:** Uchenna Okereafor, Elizabeth Makhatha, Lukhanyo Mekuto, Vuyo Mavumengwana

**Affiliations:** a Department of Metallurgy, School of Mining, Metallurgy and Chemical Engineering, Faculty of Engineering and the Built Environment, University of Johannesburg, South Africa; b Department of Chemical Engineering, School of Mining, Metallurgy and Chemical Engineering, Faculty of Engineering and the Built Environment, University of Johannesburg, South Africa; c Department of Biotechnology & Food Technology, University of Johannesburg, South Africa

**Keywords:** Mine tailings, Trace metal, Farming, Pollution, Contamination factor, Geoaccumulation index

## Abstract

Food security remains an important aspect of human lives and the vital role of soil in the global agricultural and food crops production is obvious. The quality of agricultural products which is being consumed by human through the food chain is dependent on the condition of the soil. Previous gold mining activities resulted in the discharge of tailing materials containing various hazardous trace metals such as manganese (Mn), nickel (Ni), arsenic (As), cadmium (Cd), cobalt (Co), copper (Cu), chromium (Cr), lead (Pb), and zinc (Zn). 20 representative soil samples were collected from the Gold one Mine tailing dump located in Ekuhurleni, Gauteng Province, South Africa and used in describing the prevalence and concentrations of selected trace metals using inductively coupled plasma optical emission spectrometry (ICP-OES). The concentration of identified trace metals in decreasing order is as follows: Cr > Al > As > Fe > Pb > Co > Ni > Ti > Cd > Zn > Cu. Contamination levels of trace metals in the soils were evaluated using various pollution indices such as contamination factor, degree of contamination, geo-accumulation index, pollution load index and the United States Environmental Protection Agency. These evaluations revealed a high degree and the ultra-high degree of contamination classes of soils. Based on the observed concentrations of trace metals and contamination levels, this study strongly support the call for analysis of the nearby stream and drinking water quality, including the staple crops that are cultivated within the vicinity of the dump site, to ascertain the levels of heavy metals within such crops. Stringent mitigation plans or conversion of the tailing dump into value-added products should be considered.

**Table:** Specifications Table

Subject	Environmental pollution
Specific subject area	Soil pollution and monitoring
Type of data	Table and Figure
How data were acquired	Samples were obtained from around the gold tailing dump in Ekuhurleni following prescribed sampling procedures and transferred to the laboratory. Analysing of trace metals was done using ICP-OES.
Data format	Raw and processed,
Experimental factors	Sampling the designated locations around the dump site for determination of soil characteristics and analysing trace and heavy metals concentration.
Experimental features	Determination of soil characteristics and the concentration levels of trace and heavy metals. Assessment of pollution levels using various indices such as contamination factor, degree of contamination, geo-accumulation index, pollution load index and the United States Environmental Protection Agency.
Data source location	Medical Geology Research Center, Department of Metallurgy, School of Mining, Metallurgy and Chemical Engineering, Faculty of Engineering and the Built Environment, University of Johannesburg, South Africa.
Data accessibility	Data are presented in the article.

**Table:** 

**Value of the Data**
• This data presents heavy metal contaminations in soil of a farming area located within the proximity of an abandoned mine dump.
• Farmers, government agencies, individuals as well as academic researchers stand to benefit by understanding potential dangers to the surrounding environment and humans in general emanating from abandoned mine dump sites.
• The data can be used to determine the extent and impact of toxic metals on plants and animals within farming communities.
• The data serves as a reference material in comparison with similar areas and for future scientific research for the planning, design and development of mitigation techniques.

## 1 Data

Abandoned mine tailing dumps have remained a global subject of discuss in the field of mining, metallurgy and the built environment. South Africa lies on the southernmost part of the African continent, and is known to have renowned varied topography, great natural beauty, and cultural diversity. It is a medium-sized country, with a total land area of 1,219,090 square kilometres. Ekurhuleni falls within the East Rand region in the Gauteng province and is characterized by rainfall known to be typical to the Highveld summer rainfall, which occurs from October to April. The average annual rainfall varies from 715 mm to 735 mm an indication that the study area has a distinct moisture deficit.

**Fig. 1:**
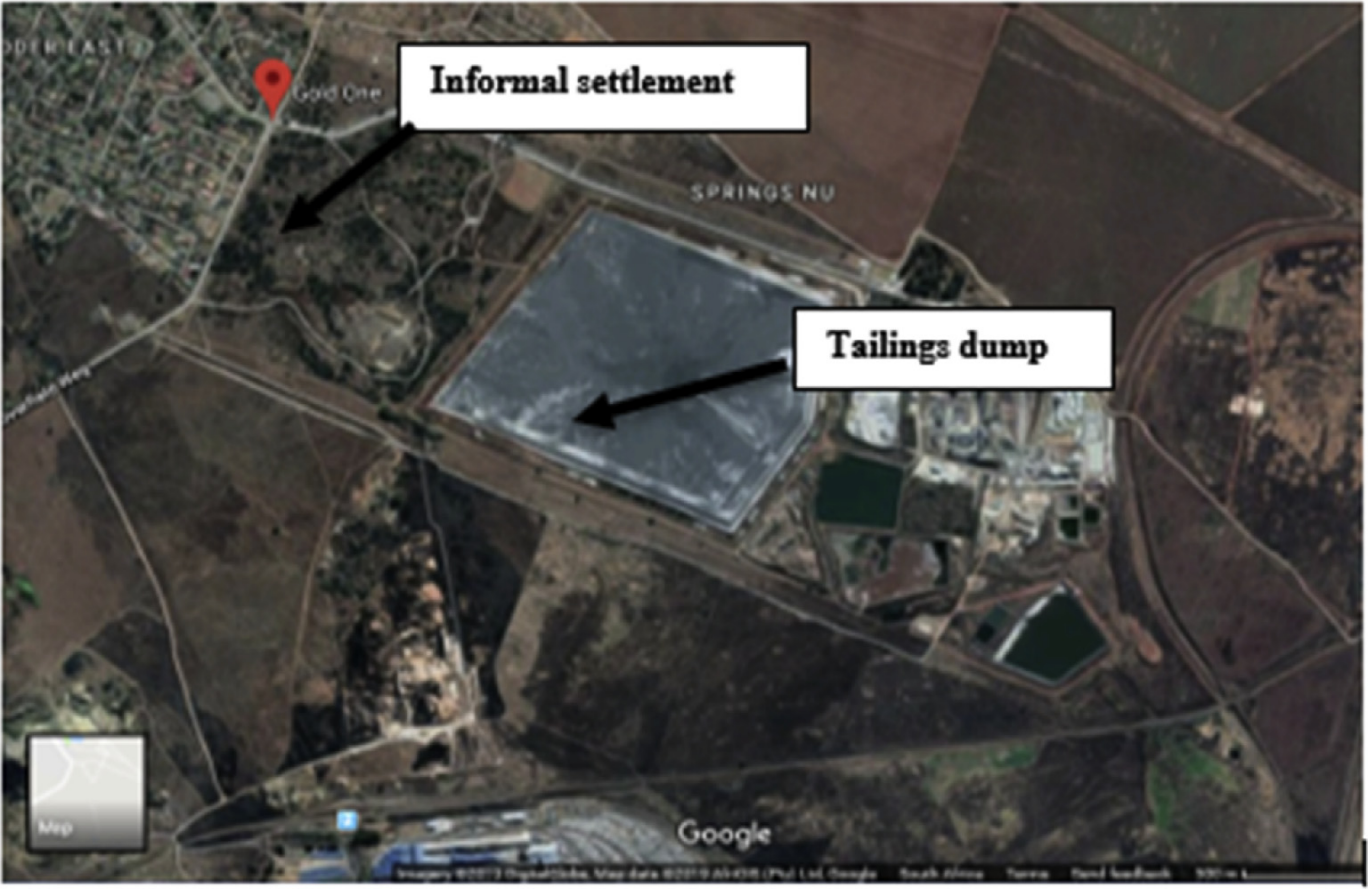
Location of the sampling site.

The data provided here is targeted towards monitoring of certain trace metals such as Cr, Al, As, Fe, Pb, Co, Ni, Ti, Cd, Zn, and Cu in the mining town of Blesbokspruit, Ekuhurleni, Gauteng province, South Africa. Fig. 1 shows the study area while Table 1 describes the locations of the gold mine tailing dump sediment samples. Presented in Table 2, Table 3, Table 4, are the terminologies used to describe contamination factor, contamination degree, and geo-accumulation index respectively. The United State Environmental Protection Agency (USEPA) guidelines for sediments in comparison with gold mine tailing dump sediments are presented in Table 5. Sieve analysis and geochemical properties of soil from gold mine tailing dump are shown in Table 6, Table 7. Trace metal concentrations, Contamination factor (CF) and Degree of contamination, Geo-accumulation index (I_geo_) and Pollution load index (PLI) of soils from gold mine tailing dump were presented in Table 8, Table 9, Table 10.

**Table 1:** Locations of the gold mine tailing sediment samples.

Station No.	Latitude (S)	Longitude (E)
1	26° 10^ʹ^	28° 27^ʹ^
2	26° 15^ʹ^	28° 35^ʹ^
3	26° 04^ʹ^	28° 40^ʹ^
4	26° 17^ʹ^	28° 44^ʹ^
5	26° 21^ʹ^	28° 50^ʹ^
6	26° 30^ʹ^	29° 10^ʹ^
7	26^0^ 00^ʹ^	29° 15^ʹ^
8	26° 27^ʹ^	29° 20^ʹ^
9	26^0^ 09^ʹ^	29° 35^ʹ^
10	26° 38^ʹ^	29° 42^ʹ^
11	26° 43^ʹ^	29° 47^ʹ^
12	26° 34^ʹ^	29° 50^ʹ^
13	26° 13^ʹ^	29° 53^ʹ^
14	26° 19^ʹ^	30° 10^ʹ^
15	26° 48^ʹ^	30° 15^ʹ^
16	26° 36^ʹ^	30° 25^ʹ^
17	26° 40^ʹ^	30° 29^ʹ^
18	26° 14^ʹ^	30° 35^ʹ^
19	26° 23^ʹ^	30° 40^ʹ^
20	26° 54^ʹ^	30° 48^ʹ^

**Table 2:** Terminologies used to describe contamination factor [8].

CF	Description
CF < 1	Low contamination factor
1 ≤ CF < 3	Moderate contamination factor
3 ≤ CF < 6	Considerate contamination factor
CF ≥ 6	Very high contamination factor

**Table 3:** Terminologies used to describe contamination degree for soil [9].

CD	Description
CD < 6	Low contamination degree
6 ≤ CD < 12	Moderate contamination degree
12 ≤ CD < 24	Considerate contamination degree
CD ≥ 24	Very high contamination degree

**Table 4:** Classification for the geo-accumulation index (Igeo) [10].

I_geo_ Value	Class	Contamination Level
I_geo_ ≤ 0	0	Uncontaminated
0 < I_geo_ < 1	1	Uncontaminated/moderately contaminated
1 < I_geo_ < 2	2	Moderately contaminated
2 < I_geo_ < 3	3	Moderately/strongly contaminated
3 < I_geo_ < 4	4	Strongly contaminated
4 < I_geo_ < 5	5	Strongly/extremely contaminated
5 < I_geo_	6	Extremely contaminated

**Table 5:** USEPA Guidelines for sediments (mg/kg dry weights).

Metal	Not polluted	Moderately polluted	Heavily polluted	Present study
Cd	–	–	>6	7.1
Cr	<25	25–75	>75	860.3
Cu	<25	25–50	>50	0.1
Pb	<40	40–60	>60	121.9
Zn	<90	90–200	>200	3.9

**Table 6:** Sieve analysis of soil from gold mine tailing dump.

Sample No.	Sieve size (ASTM) % Materials; Retains (gms)								
	No. 100	No. 140	No. 200	No. 270	PAN	TOTAL	% Sand	% Silt	% Clay
1	5.68	45.51	15.84	10.25	22.72	100	67.03	10.25	22.72
2	5.75	46.82	13.79	10.58	23.41	100	66.01	10.58	23.41
3	5.40	46.52	13.61	10.62	23.85	100	65.53	10.62	23.85
4	5.37	45.84	14.71	11.25	22.83	100	65.92	11.25	22.83
5	5.42	45.93	13.93	11.81	22.91	100	65.28	11.81	22.91
6	5.39	47.88	13.01	11.20	22.52	100	66.28	11.20	22.52
7	5.42	48.23	11.87	10.78	23.70	100	65.52	10.78	23.70
8	5.88	46.38	13.42	10.44	23.88	100	65.68	10.44	23.88
9	5.94	46.82	13.00	10.32	23.92	100	65.76	10.32	23.92
10	5.66	44.46	16.15	10.58	23.15	100	66.27	10.58	23.15
11	5.86	47.20	14.22	9.88	22.84	100	67.28	9.88	22.84
12	5.42	45.30	15.83	11.32	22.13	100	66.55	11.32	22.13
13	5.38	45.92	13.68	11.84	23.18	100	64.98	11.84	23.18
14	5.62	46.34	13.74	10.68	23.62	100	65.70	10.68	23.62
15	5.48	46.82	13.81	10.31	23.58	100	66.11	10.31	23.58
16	5.23	46.92	14.81	10.22	22.82	100	66.96	10.22	22.82
17	5.98	48.22	11.62	11.69	22.49	100	65.82	11.69	22.49
18	5.36	48.80	11.78	11.38	22.68	100	65.94	11.38	22.68
19	5.92	48.24	11.34	11.75	22.75	100	65.50	11.75	22.75
20	5.68	47.36	13.37	10.94	22.65	100	66.41	10.94	22.65

**Table 7:** Geochemical properties of soil from gold mine tailing dump.

Station No.	pH	C.E (mS/cm)	CEC (meq/100 g)	LOI (%)
1	3.86	1.30	8.5	5.1
2	4.34	1.50	8.8	5.4
3	4.28	1.80	9.0	5.0
4	4.30	1.90	8.3	5.1
5	3.92	1.40	9.1	5.3
6	4.34	1.60	8.8	5.1
7	3.89	1.40	8.5	5.4
8	3.87	1.40	9.1	5.1
9	3.86	1.40	9.0	5.2
10	4.27	1.80	8.8	5.2
11	4.28	1.80	9.4	5.4
12	4.28	1.80	8.5	5.1
13	3.88	1.40	9.3	5.2
14	3.86	1.40	8.7	5.2
15	4.30	1.60	8.3	5.4
16	3.87	1.40	9.1	5.1
17	3.86	1.40	9.0	5.1
18	4.31	1.50	8.5	5.2
19	4.27	1.90	8.8	5.1
20	4.28	1.80	9.3	5.2

**Table 8:** Trace metal concentrations (mg/kg dry weight) in soil from gold mine tailing dump.

Station No.	Cr	Al	As	Fe	Pb	Co	Ni	Ti	Cd	Zn	Cu
1	862.6	327.4	201.7	134.1	125.6	28.4	26.1	9.0	9.2	4.7	0.6
2	860.4	327.9	203.4	136.2	122.9	30.2	25.3	8.3	8.8	4.0	0.1
3	861.3	328.0	202.9	133.7	123.1	29.5	26.4	9.2	8.1	4.1	0.2
4	862.4	328.4	202.4	130.1	124.7	28.8	24.7	8.1	7.9	3.9	0.2
5	862.1	326.5	202.1	132.5	121.9	29.6	23.8	8.7	7.2	5.6	0.3
6	861.5	325.7	201.7	134.9	122.1	29.3	25.1	7.9	8.3	4.3	0.1
7	860.6	324.9	203.0	135.3	123.5	28.7	25.7	8.5	7.5	5.2	0.1
8	861.1	328.1	201.9	135.1	123.2	29.2	26.3	9.0	7.9	4.9	0.3
9	860.7	327.9	202.6	135.9	124.1	27.5	26.8	9.2	9.0	4.2	0.1
10	860.3	326.3	202.1	132.7	124.8	29.1	25.2	8.1	8.5	5.1	0.1
11	860.6	325.4	201.7	136.0	122.3	27.3	25.7	8.3	7.1	5.3	0.1
12	861.0	326.7	200.9	131.8	122.5	27.9	23.9	8.7	8.7	5.0	0.2
13	862.1	326.1	201.2	135.9	124.9	28.7	24.3	7.6	8.3	5.5	0.6
14	860.5	327.9	201.4	134.1	123.1	28.3	26.0	9.1	9.1	4.5	0.1
15	862.5	328.2	203.0	133.7	122.7	28.0	25.8	9.2	8.3	5.0	0.2
16	862.3	326.3	202.6	134.9	125.8	29.1	24.6	8.3	8.5	5.3	0.3
17	862.4	325.9	201.5	133.5	123.7	29.5	24.2	8.0	8.1	4.1	0.1
18	861.9	327.4	202.3	134.2	125.1	29.7	26.5	7.2	8.0	4.8	0.4
19	861.6	326.1	201.9	134.9	124.3	28.1	25.7	8.4	8.5	4.4	0.5
20	862.0	325.6	203.1	135.7	125.3	28.4	26.3	8.7	8.8	4.6	0.1
Mean	861.5	326.8	202.2	134.3	123.7	28.8	25.4	8.5	8.3	4.5	0.2
Max	862.6	328.4	203.4	136.2	125.8	30.2	26.8	9.2	9.2	5.6	0.6
Min	860.3	324.9	200.9	130.1	121.9	27.3	23.8	7.2	7.1	3.9	0.1
B_n_	90	88,000	13	47,200	20	19	50	4600	0.3	95	45
ISQG	52.3	NA	7.24	NA	30.2	NA	NA	NA	0.7	124.0	18.7

**Table 9:** Contamination factor (CF) and Degree of contamination of soil from gold mine tailing dump.

Station No.	Contamination factor of single metal							Degree of contamination	
	Cr	As	Pb	Co	Ni	Cd	Zn		
1	9.58	15.52	6.28	1.49	0.52	30.67	0.05	64.11	Very high
2	9.56	15.65	6.15	1.59	0.51	29.33	0.04	62.83	Very high
3	9.57	15.61	6.16	1.55	0.53	27.00	0.04	60.46	Very high
4	9.58	15.57	6.24	1.52	0.49	26.33	0.04	59.77	Very high
5	9.58	15.55	6.10	1.56	0.48	24.00	0.06	57.33	Very high
6	9.57	15.52	6.11	1.54	0.50	27.67	0.05	60.96	Very high
7	9.56	15.62	6.18	1.51	0.51	25.00	0.05	58.43	Very high
8	9.57	15.53	6.16	1.54	0.53	26.33	0.05	59.71	Very high
9	9.56	15.58	6.21	1.45	0.54	30.00	0.04	63.38	Very high
10	9.56	15.55	6.24	1.53	0.50	28.33	0.05	61.76	Very high
11	9.56	15.52	6.12	1.44	0.51	23.67	0.06	56.88	Very high
12	9.57	15.45	6.13	1.47	0.48	29.00	0.05	62.15	Very high
13	9.58	15.48	6.25	1.51	0.49	27.67	0.06	61.04	Very high
14	9.56	15.49	6.16	1.49	0.52	30.33	0.05	63.60	Very high
15	9.58	15.62	6.14	1.47	0.52	27.67	0.05	61.05	Very high
16	9.58	15.58	6.29	1.53	0.49	28.33	0.06	61.86	Very high
17	9.58	15.50	6.19	1.55	0.48	27.00	0.04	60.34	Very high
18	9.58	15.56	6.26	1.56	0.53	26.67	0.05	60.21	Very high
19	9.57	15.53	6.22	1.48	0.51	28.33	0.05	61.69	Very high
20	9.58	15.62	6.27	1.49	0.53	29.33	0.05	62.82	Very high
Average	9.57	15.55	6.19	1.51	0.51	27.63	0.05	61.01	Very high

**Table 10:** Geo-accumulation index (Igeo) and Pollution load index (PLI) of soil from gold mine tailing dump.

Station No.	Cr	As	Pb	Co	Ni	Cd	Zn	PLI	Description of PLI
1	1.85	2.34	1.43	0.00	−1.05	3.02	−3.51	2.72	Polluted
2	1.85	2.34	1.41	0.06	−1.09	2.97	−3.51	2.63	Polluted
3	1.85	2.34	1.41	0.04	−1.05	2.89	−3.51	2.61	Polluted
4	1.85	2.34	1.43	0.01	−1.11	2.87	−3.51	2.56	Polluted
5	1.85	2.34	1.40	0.04	−1.14	2.77	−3.22	2.67	Polluted
6	1.85	2.34	1.40	0.03	−1.11	2.91	−3.51	2.67	Polluted
7	1.85	2.34	1.42	0.01	−1.08	2.81	−3.22	2.64	Polluted
8	1.85	2.34	1.41	0.02	−1.05	2.87	−3.51	2.68	Polluted
9	1.85	2.34	1.42	−0.04	−1.02	3.00	−3.51	2.63	Polluted
10	1.85	2.34	1.43	0.02	−1.08	2.94	−3.22	2.68	Polluted
11	1.85	2.34	1.41	−0.04	−1.08	2.76	−3.22	2.66	Polluted
12	1.85	2.33	1.41	−0.02	−1.14	2.96	−3.22	2.65	Polluted
13	1.85	2.34	1.43	0.01	−1.14	2.91	−3.22	2.73	Polluted
14	1.85	2.34	1.41	−0.01	−1.05	3.01	−3.51	2.71	Polluted
15	1.85	2.34	1.41	−0.02	−1.08	2.91	−3.22	2.67	Polluted
16	1.85	2.34	1.43	0.02	−1.11	2.94	−3.22	2.75	Polluted
17	1.85	2.34	1.42	0.04	−1.14	2.89	−3.51	2.57	Polluted
18	1.85	2.34	1.43	0.04	−1.05	2.88	−3.51	2.69	Polluted
19	1.85	2.34	1.42	−0.01	−1.08	2.94	−3.51	2.68	Polluted
20	1.85	2.34	1.43	0.00	−1.05	2.97	−3.51	2.71	Polluted

## 2 Experimental design, materials, and methods

### 2.1 Sampling procedure

To assess the level of trace metal contamination in the soil, about 2 kg of 20 representative soil samples were obtained from the dump site which currently serves as an informal settlement for over 200 individuals. Preceding the removal of top tailing samples (2 cm) using an auger cleaned with 70% ethanol, soil samples were taken at a depth of 0–20 cm for every 50 m interval. The collected soil samples (tailing) were kept cool in an icebox (<4 °C) and transported to the laboratory for further analyses in sterile plastic bags, pre-treated with 70% ethanol to remove any traces of heavy metal contaminants. Using a GPS gadget, the precise location of each sample point was determined.

### 2.2 Analytical methods

Tailing samples were oven dried at 100 °C for 24 hours and passed through a 2 mm sieve. Aliquots of approximately 2 g of the various tailing samples were weighed into a Teflon crucible and moistened with 100 mL of 1 M HCl acid for the determination of the HCl-soluble fraction of trace metals. The mixtures were covered and placed on a shaker for 12 hours at 130 rpm. The solutions were filtered through a Whatmann filter paper, and the filtrates were stored in sterile bottles prior to analysis of metals using inductively coupled plasma-optical emission spectrometry (ICP-OES).

The trace metals were determined using ICP-OES (Model - GBC Quantima Sequential) operated under specific conditions of 1300 W RF power, 15 L min^−1^ plasma flow, 2.0 L min^−1^ auxiliary flow, 0.8 L min^−1^ nebulizer flow, 1.5 mL min^−1^ sample uptake rate. Metal determination was done using Axial view, while 2-point background correction and 3 replicates were employed in the measurement of analytical signal. The emission intensities were determined for the most sensitive lines free of spectral interference. By diluting the stock multi-elemental standard solution (1000 mg L^−1^) in 0.5% (v/v) nitric acid, the calibration standards were prepared. The calibration curves for all the studied elements were in the range of 0.01–1.0 mg L^−1^.

Physicochemical properties such as pH and EC (electrical conductivity) of the soil samples (tailing) were measured in a soil-to-water suspension (1:2.5, w/w) and a 1:5 tailings-to-water suspension using a Crison multimeter (model MM 41) respectively [1]. The grain size distribution of tailing samples was determined using the hydrometer method [2].

### 2.3 Soil pollution assessment

The level of trace metal pollution in an environment can be ascertained from the surrounding sediments by comparing the pollutant metal concentration with an unpolluted reference material. Thus, the average shale concentration as an International standard reference for unpolluted sediment was utilised [3]. This study applied pollution indices such as (i) metal contamination factor, (ii) contamination degree, (iii) index of geo-accumulation, and (iv) pollution load index using Eqs. (1), (2), (3), (4) [4], [5], [6], [7].

(1) $$\text{CF}=\frac{Mean\;metal\;concentration\;at\;contaminated\;site\;\left(Cm\right)}{Level\;of\;pre-industrial\;concentration\;of\;individual\;metal\;\left(Cbackground\right)}$$

(2) $$\text{CD}=\overset{n}{\underset{i=0}{\sum }}\text{Cf}$$

(3) $${\text{I}}_{\text{geo}}={\text{log}}_{2}\frac{\text{Cn}}{1.5\text{Bn}}$$

(4) $${\text{PLI}=\text{(}{\text{CF}}_{1}{\text{xCF}}_{2}{\text{xCF}}_{3}\text{x}\ldots {\text{xCF}}_{\text{n}}\text{)}}^{1\text{/n}}$$

where Cn is the measure of the metal concentration in the examined metal n in the sediment, Bn is the background concentration of the element (average shale concentration) or reference value of the metal n, and 1.5 is the correction factor due to the lithogenic effect that could result in variations in the background values for a given metal in the environment.

## References

[1] Shafie N.A., Aris A.Z., Zakaria M.P., Haris H., Lim W.Y., Isa N.M. (2013). Application of geoaccumulation index and enrichment factors on the assessment of heavy metal pollution in the sediments. J. Environ. Sci. Health Part A.

[2] ASTM D422-63 (2007). Standard Test Method for Particle-Size Analysis of Soils.

[3] Turekian K.K., Wedepohl D.H. (1961). Distribution of the elements in some major units of the earth's crust. Geol. Soc. Am. Bull..

[4] Cabrera F., Clemente L., Barrientos D.E. (1999). Heavy metal pollution of soils affected by the guadiamar toxic flood. Sci. Total Environ..

[5] Angula E. (1996). The tomlinson pollution index applied to heavy metal, Mussel-Watch data: a useful index to assess coastal pollution. Sci. Total Environ..

[6] Tomlinson D.L., Wilson J.G., Harris C.R., Jeffrey D.W. (1980). Problems in the assessment of Heavy metal levels in estuaries and the formation of a pollution index. Helgol. Meeresunters..

[7] Muller G. (1969). Index of geo-accumulation in sediments of the rhine river. Geojournal.

[8] Hakanson L. (1980). An ecological risk index for aquatic pollution control. A sedimentological approach. Water Res..

[9] Ahdy H.H., Khaled A. (2009). Heavy metals contamination in sediments of the western part of Egyptian mediterranean sea. Aust. J. Basic Appl. Sci..

[10] Müller G. (1969). Index of geoaccumulation in sediments of the rhine river. Geol. J..

